# Frequency of Sleep Disorders in Rheumatoid Arthritis Patients

**DOI:** 10.7759/cureus.72417

**Published:** 2024-10-26

**Authors:** Amna Yaseen, Saima Yasin, Zia Ul Haq, Iftikhar Ahmad, Manzoor Hussain, Naveed Pervez

**Affiliations:** 1 General Medicine, Russells Hall Hospital, Dudley, GBR; 2 Pulmonology, Hayatabad Medical Complex, Peshawar, PAK; 3 Pathology, Hayatabad Medical Complex, Peshawar, PAK; 4 Rheumatology, Pakistan Institute of Medical Sciences, Islamabad, PAK; 5 Gastroenterology, Rehman Medical Institute, Peshawar, PAK; 6 General Practice, Abu Dhabi National Oil, Abu Dhabi, ARE; 7 Internal Medicine, Medical A Unit, Mardan Medical Complex (MMC), Mardan, PAK; 8 Pulmonology, Khalifa Gul Nawaz Teaching Hospital, Bannu Medical College Medical Teaching Institute, Bannu, PAK

**Keywords:** disease activity, insomnia, quality of life, rheumatoid arthritis, sleep disorders

## Abstract

Background: The quality of life of people with rheumatoid arthritis (RA), a chronic inflammatory illness, is greatly impacted by sleep disturbances that are often experienced in conjunction with the condition.

Objective: The purpose of this research was to ascertain the prevalence of sleep problems in RA patients as well as their relationship to patient demographics and disease severity.

Methodology: A cross-sectional study was conducted from January to December 2023. The Pittsburgh Sleep Quality Index (PSQI) and Epworth Sleepiness Scale (ESS) were used to evaluate sleep disturbances and structured questionnaires were used to gather data. Demographic characteristics and the severity of the RA condition were also assessed. using a significance threshold set at p<0.05, Chi-square tests were used to evaluate the relationship between sleep problems and the severity of RA illness in the statistical studies carried out using IBM SPSS Statistics for Windows, version 26 (IBM Corp., Armonk, NY, USA).

Results: A total of 385 RA patients were included in the study. Among them, 38.96% (n=150) reported insomnia, 18.18% (n=70) suffered from sleep apnea, and 15.58% (n=60) experienced restless legs syndrome. Poor sleep quality was prevalent in 75.32% (n=290) of participants, as assessed by the PSQI. Notably, a significant association was found between insomnia and moderate disease activity (n=55; p<0.001), while sleep apnea was most frequently observed in patients with low disease activity (n=27; p=0.003). Additionally, restless legs syndrome was predominantly seen in those with moderate disease activity (n=26; p=0.002), highlighting the complex relationship between RA severity and sleep disturbances.

Conclusion: The high prevalence of sleep disorders in RA patients, particularly insomnia and sleep apnea, is significantly associated with disease severity, highlighting the need for targeted management strategies.

## Introduction

Rheumatoid arthritis (RA) is a chronic inflammatory illness that primarily affects the joints, causing pain, stiffness, and disability [1,2]. However, the impact of RA extends beyond the musculoskeletal system; many patients also suffer from comorbid conditions that significantly affect their quality of life [3]. One of the most common yet often underrecognized comorbidities is sleep disturbance [4]. RA’s inflammatory processes, pain, and psychological stress contribute to disrupted sleep, creating a complex interaction between disease symptoms and sleep quality [5].

Common sleep disorders in the general population include insomnia, apnea, and restless legs syndrome, but RA patients appear to be disproportionately affected by these issues [6]. Studies have shown that more than 50-75% of RA patients suffer from sleep disturbances, although the exact causes remain unclear [7-9]. Pain, especially nocturnal discomfort linked to RA, may play a significant role in disrupting sleep patterns [9]. Furthermore, recent research has demonstrated that inflammation plays a direct role in sleep regulation, increasing the vulnerability of RA patients to a variety of sleep disturbances, including insomnia and sleep apnea [10].

Sleep disturbances exacerbate the overall burden of RA. Poor sleep not only worsens daytime functioning but also heightens pain sensitivity and impairs overall bodily function, creating a feedback loop that can aggravate RA disease activity [11]. Chronic sleep deprivation is believed to maintain a state of elevated inflammation, further accelerating disease progression and worsening the patient’s condition [12].

Despite individual studies exploring the association between RA and sleep, there is limited knowledge regarding the specific types and frequencies of sleep disorders experienced by RA patients across different demographic groups. Understanding the extent of sleep disturbances in this population is critical for improving both treatment strategies and overall patient outcomes.

Research objective

This study aims to determine the prevalence of sleep disturbances in RA patients and their correlation with patient demographics and disease severity.

## Materials and methods

Study design and setting

This cross-sectional study was conducted at the Pakistan Institute of Medical Sciences (PIMS) in Islamabad and Khalifa Gul Nawaz Teaching Hospital in Bannu from January 2023 to December 2023. These institutions were selected for their diverse patient populations and established rheumatology departments, which provide comprehensive care to individuals diagnosed with rheumatoid arthritis (RA).

Inclusion and exclusion criteria

Adult patients aged 18 years or older with a confirmed diagnosis of rheumatoid arthritis, as defined by the American College of Rheumatology's (ACR) categorization criteria, were eligible for inclusion in the study. To ensure consistency in treatment evaluation, patients had to have been on rheumatoid arthritis therapy for a minimum of six months prior to enrollment.

Patients were excluded from the study if they were on medications known to significantly affect sleep patterns, such as sedatives or antidepressants, or if they had other major comorbidities, including but not limited to heart failure or chronic obstructive pulmonary disease (COPD). This was done to eliminate confounding variables that could potentially skew the results regarding sleep disturbances in the RA population.

Sample size

The sample size was calculated to ensure adequate power for statistical analysis. Using a 95% confidence interval and a 5% margin of error, we based our calculation on an anticipated 50% prevalence of sleep disorders among patients with RA, as reported in previous literature and endorsed by the World Health Organization (WHO) for prevalence studies. Consequently, a total of approximately 385 individuals were recruited to participate in the study, allowing for comprehensive data analysis while accounting for potential dropouts.

Data collection

Data were collected using a standardized questionnaire designed to evaluate various aspects of the study. The questionnaire assessed the occurrence of sleep disturbances, and the severity of rheumatoid arthritis using the Disease Activity Score 28 (DAS28), and pertinent demographic information, including age, gender, duration of disease, and treatment history.

The quality of sleep was measured using validated scales: the Pittsburgh Sleep Quality Index (PSQI) [13] and the Epworth Sleepiness Scale (ESS) [14]. These tools are well-established for evaluating sleep quality and daytime sleepiness, respectively, and have been widely used in previous studies. Trained medical staff conducted in-person interviews with patients to administer the questionnaire, ensuring accurate data collection and the opportunity to clarify any questions.

Statistical analysis

Data were analyzed using IBM SPSS Statistics for Windows, version 26 (IBM Corp., Armonk, NY, USA). Descriptive statistics were employed to summarize clinical features and patient demographics. The relationship between sleep disturbances and the severity of rheumatoid arthritis was evaluated using Chi-square tests. The frequency of sleep abnormalities was computed, and p-values less than 0.05 were considered statistically significant. Additionally, multivariate analysis was conducted to control for potential confounders, providing deeper insights into the factors influencing sleep quality among patients with rheumatoid arthritis.

Ethical approval

The study received ethical clearance from the Institutional Review Board (IRB) of both participating hospitals. Before enrollment, all participants provided written informed consent, ensuring they were aware of the study's objectives and their rights regarding participation. This process adhered to ethical guidelines for conducting research involving human subjects.

## Results

There were 385 rheumatoid arthritis (RA) patients in the research; the majority were over 60 (110; 28.57%), followed by those between 51 and 60 (100; 25.97%). Significantly more women than men made up the sample (292; 75.79%), with 93 (24.21%) being men. The majority of participants (82.05%; 316) were married, whereas the percentage of single people (15.58%) and widowed people (2.34%; 9) was lower. In terms of education, 40 (10.39%) had no formal education, 187 (48.57%) had a higher education, and 98 (25.45%) had a secondary education. The majority (251; 65.19%) lived in cities. Regarding the length of RA, 215 patients (55.84%) had it for more than five years, 120 patients (31.17%) for one to five years, and 50 patients (13.00%) for less than a year (Table 1).

**Table 1:** Demographic information of participants RA: rheumatoid arthritis

Characteristic		Number of patients (n;%)
Age (years)	18-30	30 (7.79)
	31-40	55 (14.29)
	41-50	90 (23.38)
	51-60	100 (25.97)
	>60	110 (28.57)
Gender	Male	93 (24.21)
	Female	292 (75.79)
Marital status	Single	60 (15.58)
	Married	316 (82.05)
	Widowed	9 (2.34)
Education level	No formal education	40 (10.39)
	Primary education	60 (15.58)
	Secondary education	98 (25.45)
	Higher education	187 (48.57)
Residence	Urban	251 (65.19)
	Rural	134 (34.81)
Duration of RA (years)	<1 year	50 (13.00)
	1-5 years	120 (31.17)
	>5 years	215 (55.84)

Table 2 displays the DAS28-assessed RA disease severity for a group of 385 patients: Of the 80 participants, 20.78% were in remission, 31.17% had low disease activity, 36.36% had moderate disease activity, and 11.69% had severe disease activity.

**Table 2:** RA disease severity as assessed by DAS28 RA: rheumatoid arthritis; DAS28: Disease Activity Score 28

DAS28 severity category	DAS28 score range	Number of patients (n;%)
Remission	< 2.6	80 (20.78)
Low disease activity	2.6 - 3.2	120 (31.17)
Moderate disease activity	3.2 - 5.1	140 (36.36)
High disease activity	> 5.1	45 (11.69)

**Figure 1:**
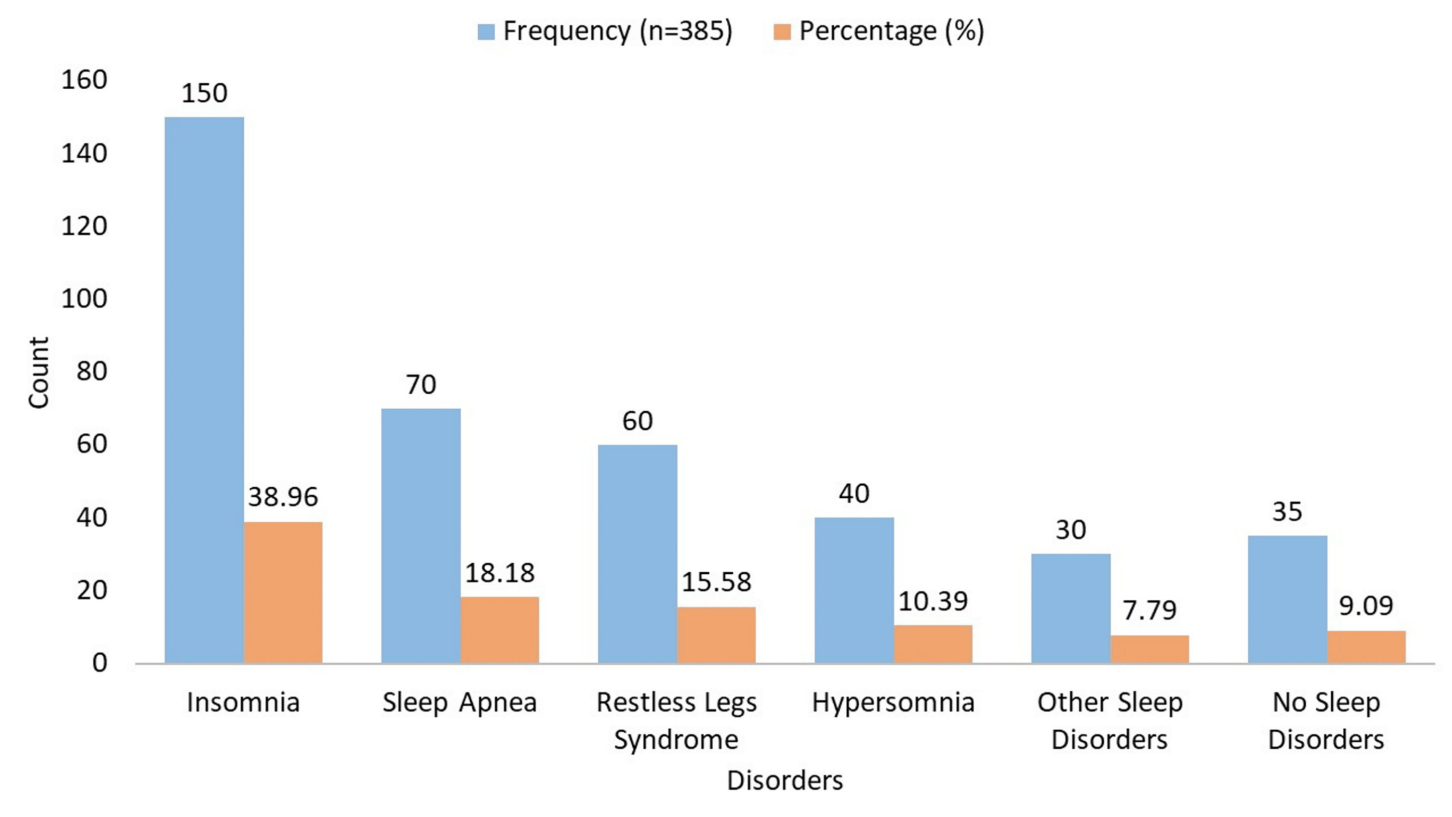
Presence of sleep disorders in RA patients RA: rheumatoid arthritis

Insomnia was reported by 38.96% of patients (n = 150) with RA, sleep apnea by 18.18% of patients (n = 70), restless legs syndrome by 15.58% of patients (n = 60), hypersomnia by 10.39% of patients (n = 40), other sleep disorders by 7.79% of patients (n = 30), and no sleep disorders by 9.09% of patients (n = 35) are depicted in Figure 1.

According to the PSQI, 24.68% (n=95) of RA patients had excellent sleep quality and 75.32% (n=290) had poor sleep quality (Table 3 summarizes the evaluation of sleep quality in these patients). Furthermore, on the ESS, 57.14% (n=220) reported normal drowsiness and 42.86% (n=165) reported extreme sleepiness.

**Table 3:** Sleep quality assessment in RA patients RA: rheumatoid arthritis

Assessment tool		Score range	Number of patients (n;%)
Pittsburgh Sleep Quality Index (0-21)	Good sleep quality	0 - 5	95 (24.68)
	Poor sleep quality	6 - 21	290 (75.32)
Epworth Sleepiness Scale (0 - 24)	Normal sleepiness	0 - 10	220 (57.14)
	Excessive sleepiness	11 - 24	165 (42.86)

Insomnia was most common in patients with moderate disease activity (n=55) and was substantially correlated with disease severity (p<0.001), according to Table 4, which looks at the relationship between sleep disturbances and the severity of RA illness. Moreover, a significant correlation was found between sleep apnea and low disease activity (n = 27, p = 0.003), with sleep apnea having the greatest prevalence in this category and restless legs syndrome (n = 26, p = 0.002). There was a significant difference in the prevalence of sleep disorders among individuals in remission (n=24, p<0.001). However, no significant correlation was observed between hypersomnia and RA disease severity (p=0.104) or between other sleep disorders (p=0.255).

**Table 4:** Association between sleep disorders and RA disease severity FRA: rheumatoid arthritis

Sleep disorder type	Remission (n=80)	Low disease activity (n=120)	Moderate disease activity (n=140)	High disease activity (n=45)	*X*^2^	p-value
Insomnia	17	48	55	30	20.42	<0.001
Sleep apnea	11	27	27	5	19.33	0.003
Restless legs syndrome	13	18	26	3	12.97	0.002
Hypersomnia	6	11	18	5	5.84	0.104
Other sleep disorders	9	7	12	2	1.84	0.255
No sleep disorders	24	9	2	0	0.001	<0.001

## Discussion

Mustafa et al. reported that sleep abnormalities affect approximately 20% of individuals with RA, with the prevalence of sleep disturbances in these patients varying widely between 18.5% and 86.5% across different studies [6,15,16]. In this research, sleep abnormalities were also common, with 18.18% (n=70) of patients reporting sleep apnea, 15.58% (n=60) experiencing restless legs syndrome, and 38.96% (n=150) suffering from insomnia. These findings align with those previous studies demonstrating a high prevalence of sleep disorders in RA patients.

The results show that 75.32% (n=290) of participants had poor sleep quality, according to the PSQI. This is consistent with earlier research, which discovered that over 70% of RA patients had poor sleep quality [17]. Since sleep quality directly influences how patients perceive pain and their general well-being, this high prevalence of poor sleep quality highlights the critical need for addressing sleep-related issues in RA patients.

A significant finding in this study was the correlation between insomnia and moderate disease activity (n=55, p<0.001). This is supported by prior studies showing that patients with higher disease activity tend to experience more sleep disturbances [18,19]. The underlying mechanism may be linked to the inflammatory processes involved in RA. Pro-inflammatory cytokines such as TNF-alpha and IL-6, which are elevated in patients with RA, interfere with sleep regulation. These cytokines increase pain sensitivity and contribute to nocturnal joint pain, making it difficult for patients to fall asleep or stay asleep. This interaction between pain and inflammation likely explains the higher prevalence of insomnia in patients with moderate disease activity.

Interestingly, sleep apnea was most common among patients with low disease activity (n=27, p=0.003). This finding is counterintuitive, as sleep disturbances are generally expected to increase with disease severity. However, this result may be explained by other contributing factors, such as obesity, which is a known risk factor for sleep apnea. Patients with low RA activity may have fewer mobility restrictions and could potentially experience weight gain, which can contribute to airway obstruction and increase the likelihood of sleep apnea. Medications commonly used in RA treatment, such as glucocorticoids, can lead to weight gain and fat redistribution, further exacerbating sleep apnea risk. This suggests that factors other than RA severity, like comorbid conditions, may play a significant role in the prevalence of sleep apnea, aligning with findings by Sutherland et al. [20].

The restless legs syndrome (RLS) findings, with a higher prevalence among patients with moderate disease activity (n=26, p=0.002), further support the connection between RA and sleep disturbances. Inflammation and neuropathic pain associated with RA can contribute to RLS, as chronic pain and nerve damage often exacerbate the symptoms of restless legs [21]. Factors like iron deficiency, which can be more common in RA patients due to chronic inflammation or dietary restrictions, may also play a role in the development of RLS. Thus, RA-related pain and discomfort likely contribute to the increased occurrence of RLS in this patient group.

These findings suggest that pain, inflammation, and sleep disturbances are interconnected in a vicious cycle. Poor sleep can lead to elevated pro-inflammatory cytokines, which in turn worsen RA symptoms, including pain and joint stiffness. This, in turn, disrupts sleep further, creating a self-perpetuating loop that exacerbates both sleep disturbances and RA disease activity. Patients with moderate disease activity, who may already be experiencing increased inflammation, may find their symptoms aggravated by sleep disturbances, perpetuating the cycle of worsening health and sleep quality.

Study limitations

This study has some limitations. The cross-sectional design limits our ability to establish causal relationships between sleep disturbances and RA severity. The use of self-reported measures for sleep quality may introduce bias, as patients may underreport or overreport their symptoms. The sample was drawn from a single center, limiting the generalizability of the results. Unmeasured confounding factors, such as comorbidities and medication effects, may have influenced the observed associations. Future research should focus on longitudinal studies with more diverse samples to better understand these interactions.

## Conclusions

The inflammatory nature of RA significantly contributes to the link between sleep disturbances and disease severity, particularly for conditions like insomnia and restless legs syndrome, which are driven by pain and inflammation. Sleep apnea, however, may be more influenced by comorbid conditions such as obesity. Effectively managing these sleep disturbances requires a holistic approach that not only targets RA-related inflammation and pain but also considers lifestyle factors such as weight management and potential nutritional deficiencies. By addressing these aspects, we can potentially break the cycle of inflammation and poor sleep, leading to improvements in both sleep quality and overall disease management. This study highlights the high prevalence of sleep disorders, especially insomnia, sleep apnea, and restless legs syndrome, among RA patients and emphasizes the intricate connection between disease activity and sleep quality. Targeting sleep disturbances could reduce the overall impact of RA, improve patient outcomes, and enhance quality of life. Future research should focus on developing tailored therapies to address these sleep issues in RA patients.
